# Single-cell RNA sequencing and binary hierarchical clustering define lung interstitial macrophage heterogeneity in response to hypoxia

**DOI:** 10.1152/ajplung.00104.2022

**Published:** 2022-05-24

**Authors:** Nzali V. Campbell, Claudia Mickael, Sushil Kumar, Hui Zhang, Ian L. Campbell, Austin E. Gillen, Caio O. Trentin, Katrina Diener, Bifeng Gao, Vitaly O. Kheyfets, Sue Gu, Rahul Kumar, Tzu Phang, R. Dale Brown, Brian B. Graham, Kurt R. Stenmark

**Affiliations:** ^1^Department of Pediatrics, University of Colorado, Anschutz Medical Campus, Aurora, Colorado; ^2^Department of Medicine, University of Colorado, Anschutz Medical Campus, Aurora, Colorado; ^3^Department of Medicine-Pulmonary Sciences & Critical Care, University of Colorado, Anschutz Medical Campus, Aurora, Colorado; ^4^School of Humanities and Science, Stanford University, Stanford, California; ^5^Division of Hematology, Department of Medicine, University of Colorado Anschutz Medical Campus, Aurora, Colorado; ^6^Department of Medicine, University of California, San Francisco, California; ^7^Department of Medicine-Bioinformatics, University of Colorado, Anschutz Medical Campus, Aurora, Colorado

**Keywords:** hypoxia, macrophages, PH, single-cell RNAseq

## Abstract

Few studies have examined lung interstitial macrophage (IM) molecular phenotypes after being exposed to hypoxia in vivo at the single-cell level, even though macrophages contribute to hypoxic pulmonary hypertension (PH). We aimed to determine IM diversity and its association with hypoxia-induced PH. We hypothesized that integrating single-cell RNA sequencing (scRNAseq) and binary hierarchal clustering (BHC) could resolve IM heterogeneity under normal homeostatic conditions and changes induced by hypoxia exposure. *Cx3cr1*^GFP/+^ reporter mice were exposed to normoxic conditions (∼21% $\text{F}{\text{I}}_{{\text{O}}_{2}}$) or exposed to 1 day (*D1*) or 7 days (*D7*) of hypoxia (∼10% $\text{F}{\text{I}}_{{\text{O}}_{2}}$). We used flow cytometry to isolate *Cx3cr1*^+^ IMs and the 10X Genomics platform for scRNAseq, Cell Ranger, Seurat, ClusterMap, monocle, ingenuity pathway analysis, and Fisher’s exact test (*q* value < 0.05) for functional investigations. *n* = 374 (normoxia), *n* = 2,526 (*D1*), and *n* = 1,211 (*D7*) IMs were included in the analyses. We identified three normoxia-related cell types, five hypoxia-associated cell types that emerged at *D1*, and three that appeared at *D7*. We describe the existence of a putative resident trained innate IM, which is present in normoxia, transiently depleted at *D1*, and recovered after 7 days of sustained hypoxia. We also define a rare putative pathogenic population associated with transcripts implicated in PH development that emerges at *D7*. In closing, we describe the successful integration of BHC with scRNAseq to determine IM heterogeneity and its association with PH. These results shed light on how resident-trained innate IMs become more heterogeneous but ultimately accustomed to hypoxia.

## INTRODUCTION

Inflammation and immune dysfunction characterize hypoxic-associated pulmonary hypertension (PH), and lung interstitial macrophages (IMs) contribute (1–3). The experimental model of hypoxia-induced PH that is based on rodent exposure to reduced oxygen levels for more than 2 wk is characterized by moderate vascular thickening. Moreover, the experimental models of hypoxia-induced PH based on rodent exposure to reduced oxygen levels are marked by the accumulation of monocytes/macrophages in the perivascular space of the lung (4, 5). The potential relevance of macrophages to hypoxia-induced vascular remodeling is supported by a study that showed that blocking the recruitment of monocytes/macrophages to the hypoxic lung markedly reduced vascular remodeling but not right ventricular hypertrophy (RVH; 6). Our group also analyzed mice exposed to 4 and 14 days of hypoxia (18,000 ft) or sea level (normoxia) and observed an accumulation of IMs after 4 days of hypoxia that decreased to baseline levels by *day 14* post hypoxia (7). Moreover, the study demonstrated that IMs after 4 days of hypoxia exposure displayed a “PH program” depicted by proinflammation gene activity, mitochondrial dysfunction, and mammalian target of rapamycin (mTOR) signaling. However, by *day 14* of hypoxia exposure, the IM population displayed a unique anti-inflammatory/pro-reparative programming state (7). These studies, however, failed to capture how hypoxia affects specific subpopulations of IMs at very early time points of hypoxia exposure (1 day or 1 wk), at the cell level, or how hypoxia affects their numbers and functional status. This study aims to fill this knowledge gap.

Macrophages belong to the cellular arm of innate immunity and are an essential source of pattern recognition molecules. They play a role in initiating, orienting, and regulating features of the adaptive response (8). During an inflammatory response, macrophages eliminate injured cells and alert the immune system through the macrophage disappearance reaction (MDR) and replenish the space through a mechanism termed the “niche model” (9, 10). However, investigations of these mechanisms failed to show the transcriptomic heterogeneity within and between macrophage populations at different time points (11). Thus, we also aimed to determine IM diversity at baseline (normoxia), *day 1* (to capture the initial effects of hypoxia on the IMs), and *day 7* to show how IMs react to prolonged hypoxia and whether any IM groups are associated with PH. We used a hypoxia exposure time-course study using single-cell RNA sequencing (scRNAseq) to highlight the transcriptome’s dynamics and heterogeneity and reveal high-resolution mechanisms of different biological processes. We used binary hierarchical clustering (BHC) to maintain this dimension, which retains time series tags, quantifies exposure, and control comparisons to track temporal population relationships (12). In other words, we used BHC because it uses the binary value of marker genes instead of the expression level. The expression of a gene is not the same between different samples, even after normalization. Therefore, the relative expression level of a gene across cells or within a cell may vary under various conditions. Thus, whether a gene is recognized as a marker of a specific subgroup or not is more robust. The binary value also tolerates the global shift of the transcriptome during matching. Thus, the binary distance used becomes more reasonable than the Euclidian distance (12). Moreover, although one could perform clustering analysis on a combined sample data set, which would increase the resolution by increasing the cell numbers of the pooled data set, it would be hard to match the subgroups in every single sample. The grouping results in BHC keep the grouping information in the individual sample data sets (12).

Our rationale was that preserving the gene expression levels within groups across different hypoxia exposure time points would provide the highest single-cell resolving power to detect rare cells representing minor cell types in an organism. Investigators have found that despite the low abundance, rare cell populations play an important role in determining the pathogenesis of the disease (13–15). We hypothesized that we could identify distinct lung IM populations in mice exposed to hypoxia that play a role in early PH development.

## MATERIALS AND METHODS

### Mice

Animal procedures were executed according to protocols approved by the University of Colorado Institutional Animal Care and Use Committee. Cx3cr1^GFP/+^ reporter mice [B6.129P2(Cg)-Cx3cr1tm1Litt/J, Jackson Labs Stock No.: 005582] were crossbred with C57BL/6J (Jackson Stock No. 000664) and raised at 1,609 m (Denver altitude) for at least four generations. At the beginning of the experiment, 5-wk-old Cx3cr1^GFP/+^ Het mice were transferred to a normoxia sea-level (SL) chamber (∼21% $\text{F}{\text{I}}_{{\text{O}}_{2}}$) for 4 wk and then hypoxia-challenged at an altitude of 5,486 m (∼10% $\text{F}{\text{I}}_{{\text{O}}_{2}}$) for 1 (*D1*) and 7 (*D7*) days at the Cardiovascular Research Core at the University of Colorado (UCD). Each time point consisted of two age and sex-matched mice (1 male and 1 female), except SL (1 female mouse). Cells from each experimental time point were pooled (16–18).

### Lung Single-Cell Isolation, Staining, Flow Cytometry, and scRNAseq Preparation

After hypoxia exposure, we sedated the mice with a ketamine/xylazine cocktail. Then we injected them retro-orbitally with a PE-labeled anti-Cd45 antibody (1 μg/mouse) 5 min before euthanasia to label the intravascular compartment. Briefly, antibodies were validated by serial titration; we checked side-scatter and forward-scatter (FWD) patterns from each population to verify if the population stained corresponded to their SSC and FWD patterns (which correspond to cell granularity and size, respectively). We used fluorescence minus one (FMO) controls to establish gating and identification of cell populations.

**Figure 1.**
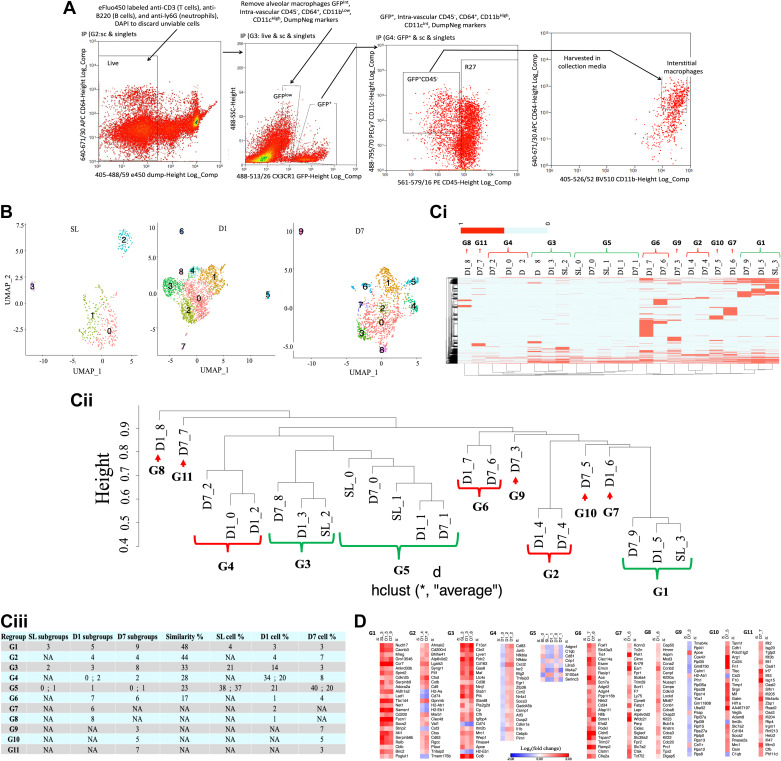
Hypoxia induces time-dependent changes in macrophage populations. *A*: gating scheme used for FACS preceding scRNAseq experiments. *B*: UMAP (uniform manifold approximation and projection for dimension reduction) plots: normoxia [sea level (SL) ∼21% $\text{F}{\text{I}}_{{\text{O}}_{2}}$] (*n* = 374), *day 1* (*D1*, *n* = 2,526), and *day 7* (*D7*, *n* = 1,211) post hypoxia exposure [5,486 m (hypoxia = ∼10% $\text{F}{\text{I}}_{{\text{O}}_{2}}$)]. *Ci*: heatmap of the marker genes for each subgroup from each experimental time point. *Cii*: dendrogram of the hierarchical clustering. Red = hypoxia-associated cell types. Green = normoxia-related cell types. *Ciii*: binary hierarchical clustering results for quantifying the subgroup comparisons. *D*: heatmap: signature genes for each group. scRNAseq, single-cell RNA sequencing; UMAP, uniform manifold approximation and projection.

The lungs were flushed with phosphate-buffered saline (PBS), harvested, and digested (19). Dump gating was used to eliminate neutrophils, B and T cells, and dead cells (Fig. 1*A*). The cells were stained using fluorochrome-conjugated antibodies (available on request). Dump gating that comprised eFluo450 labeled anti Cd3 (T cells), anti-B220 (B cells), and anti-ly6G (neutrophils) was used to eliminate neutrophils, B and T cells, and dead cells. The cells were also labeled with DAPI to discard unviable cells. Of note, interstitial macrophages were gated on green fluorescent protein (GFP)^+^, intravascular Cd45^−^, Cd64^+^, Cd11b^High^, Cd11c^Int^, and Dump^Neg^ markers (Fig. 1*A*). Alveolar macrophages, which were also eliminated were sorted using GFP^Int^, intravascular Cd45^−^, Cd64^+^, Cd11b^Low^, Cd11c^High^, and Dump^Neg^ markers. Astros EQ cell sorter (Beckman Coulter Life Sciences) at the UCD Cancer Center Flow Cytometry Shared Resource was used for cell sorting. Sorted cells were harvested in collection media (HBSS-Gibco with 2.5% FBS), centrifuged, counted, and submitted to the Genomics Core at UCD. The cells were at least 80% viable in a 700–1,000 cells/µL concentration. According to the manufacturer's instructions, we used 300 pM cDNA to prepare the libraries with the Chromium Single Cell 3' Reagent Kits v2 User Guide • Rev A to build the sequencing libraries (10X genomics platform). We used an Illumina NovaSEQ6000 to sequence the libraries.

### Read Alignment, Cell Quantification, and Quality Control

The sequenced data were processed by the Cell Ranger single-cell software suite (v2.1.1) by 10X Genomics. Briefly, raw binary base call (BCL) sequence files from an Illumina NovaSEQ6000 sequencer were demultiplexed into FASTQ format. FASTQ files from each sample were aligned and filtered, barcode and unique molecular identifiers (UMIs) counted, feature barcode matrices generated, clusters determined, and gene expression analyses and normalization performed. The sequencing reads were aligned to the mouse GRCm38/mm10 reference genomes using the STAR aligner (20).

### Single-Cell RNAseq Analysis and Visualization

Filtered feature barcode matrices from the Cell Ranger workflow were further analyzed using the Seurat R package (v.3.2.0; 21). Briefly, we followed standard practices to exclude low-quality cells. Cells with a unique feature count >4,000, <200, and >5% mitochondria content were filtered (21, 22).

Gene expression measurements were normalized and log-transformed using the global-scaling method (21). In the downstream analyses we included transcripts with high cell-to-cell variations (2,000 features per data set). Before principal component analysis (PCA) dimension reduction, we applied a standard preprocessing linear transformation (“scaling”) and performed PCA on the scaled data (21). Elbow plots selected the first 15 principal components (PCs) used in constructing a k-nearest neighbor graph based on the Euclidean distance in PCA space. The Louvain algorithm iteratively clustered cells and optimized the clusters. We used a resolution parameter of 0.5 to determine transcriptionally distinct populations. The first 15 PCs were also used as input to the uniform manifold approximation and projection (UMAP) used for dimension reduction and data visualization. We identified positive and negative differentially expressed genes (DEGs) of a subgroup compared with all other cells based on the nonparametric Wilcoxon rank-sum test (*q* value < 0.05; 21).

For binary hierarchical clustering (BHC), we used marker genes that passed the *q* value < 0.05 quality control filter from subgroups identified by Seurat as input for ClusterMap (v0.1.0) and followed the standard BHC analysis workflow (12). Briefly, hierarchical clustering analysis of the binary expression patterns of marker genes matched the most similar subgroups while preserving the gene expression levels across cells and data sets and avoiding forming large subgroups from the same data set. The Jaccard distance and average linkage are used to perform the hierarchical clustering of the binary matrix (12). Two branches were merged if >90% of the marker genes were similar. Similarity measures quantified matching quality. Whether the matched subgroups were best or not was selected relative to other subgroups. Subgroups with low similarity were unmatched (12).

### Single-Cell Trajectories

Filtered feature barcode matrices from the Cell Ranger pipeline were used as input in Monocle (v2.16.0) to infer timelines for the development of cells. We followed standard practices to exclude low-quality cells. Briefly, we used the negative binomial distribution with fixed variance to model the UMIs. Transcripts beyond ±2 standard deviations from the mean and cells with transcripts >4,000 and <200, and UMIs <2,000 and >11,000 were filtered. We included transcripts with a mean expression ≥0.1. Using the percentage of variance explained by each component based on a PCA performed on the normalized expression data, we included 25 components determined by an Elbow plot (23).

We followed the standard workflow for the trajectory analyses, used transcripts expressed in at least 50 cells, and chose genes that define progress in our data. Briefly, for the differential analysis, Monocle used vector generalized additive models to model a gene’s expression level as a smooth, nonlinear function of pseudo-time and found transcripts with an expression pattern that varies (*q* value < 0.05) according to pseudo-time (23).

### Gene Validation

We used reverse transcription-quantitative PCR (RT-qPCR) to validate transcripts related to IM function. The same conditions used to treat mice, isolate, and flow-sort cells above were repeated, except we used *n* = 3 (1 male and 2 females) at SL; *D1*, *n* = 3 (1 female and 2 males); and *D7*, *n* = 2 (1 male and 1 female). The samples were not pooled.

We used the RNeasy Micro Kit (Qiagen) to extract total RNA. Total RNA was reverse transcribed to cDNA using an iScript cDNA Synthesis Kit (Bio-Rad) for mRNA detection. The mRNA levels were determined quantitatively using quantitative-reverse transcriptase PCR (qRT-PCR). The iTaq Universal SYBR Green Supermix (Bio-Rad) and unique primer pairs (available on request) were used to detect mRNAs. We used the QuantStudio 6 Flex System (Thermo Fisher Scientific) to perform the qRT-PCR product analysis, and the delta threshold cycle method to report the results as relative expressions. Genes were compared with hypoxanthine phosphoribosyl-transferase (HPRT).

### Functional Analyses

We used ingenuity pathway analysis (IPA) for the biological annotations and Morpheus heatmaps to visualize group biomarkers, functions, pathways, and upstream regulator comparisons (Ingenuity Systems, http://www.ingenuity.com; 24).

### Statistics

We performed the Fisher’s exact test (Benjamini–Hochberg adjusted *P* value < 0.05) to determine the statistical significance of the biological annotations in IPA.

## RESULTS

### Hypoxia Induces Time-Dependent Changes in Macrophage Populations

To isolate resident and recruited IMs, we used the workflow in Fig. 1*A*. After the initial quality control using Cell Ranger, *n* = 432 (normoxia), *n* = 2,978 (*D1*), and *n* = 1,734 (*D7*) IMs were obtained. *n* = 374 (normoxia), *n* = 2,526 (*D1*), and *n * = 1,211 (*D7*) IMs were included in the final analyses after additional quality control using Seurat.

Louvain clustering of IMs identified four subgroups in normoxia, nine at *D1* and ten at *D7* (Fig. 1*B*). BHC refined the Louvain clustering based on its matching results in the next step by merging similar subgroups in the same sample (12). Subgroups were not labeled with known markers at the beginning to enable unbiased matching (12). To compare subgroups from normoxia (4), *D1* (9), and *D7* (10), BHC clustered the subgroups based on marker genes from all 23 subgroups (Fig. 1*Ci*), producing the clustering dendrogram (Fig. 1*Cii*). Next, the dendrogram was decomposed by deciding which subgroups formed matched new groups based on the similarity and purity of the subgroups. ClusterMap used the specific tree-cutting algorithm to compare the subgroups (12), resulting in 11 new groups, and computed the similarity of the matched groups (Fig. 1*Ciii*). There were no matched groups for groups (G) 7 (G7), G8, G9, G10, and G11 (Fig. 1*C*) at the edge cutoff of 0.1 (12).

Examination of the groups identified normoxia-related cell types (NRCTs) G1, G3, and G5 found not only in normoxic but also in hypoxic conditions (Fig. 1*Ciii*). Hypoxia-associated cell types (HACTs) were identified only in hypoxic conditions (Fig. 1*Ciii*). They included G2, G4, G6, G7, and G8, which appeared at *D1*, and three rare groups, G9, G10, and G11, emerged at *D7* (Fig. 1*C*, *ii* and *iii*). Evaluation of the gene expression profiles within G4 and G5 varied under different hypoxia exposure time points, whereas for G9, the gene expression profile differed from the other two rare groups at *D7* (Fig. 1*D*).

### Three Distinct IM Groups Coexist in the Normoxic Lungs

**Figure 2.**
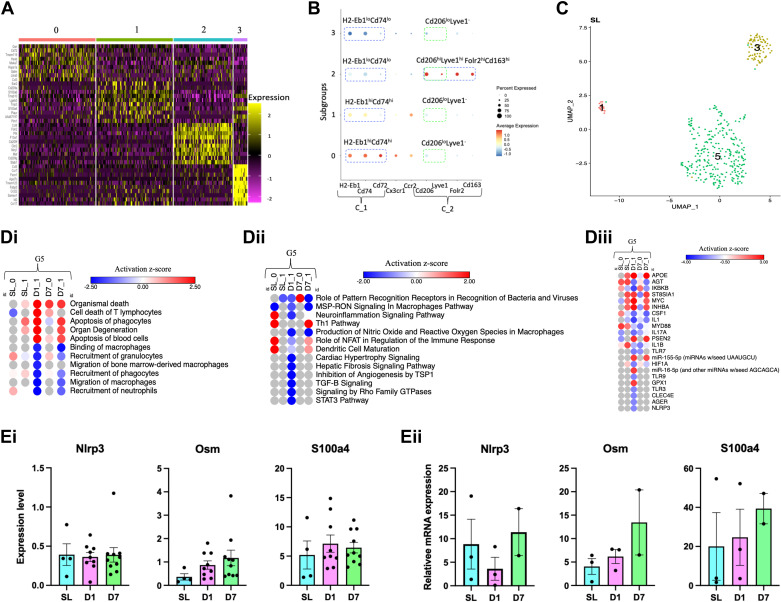
Normoxia-related cell types. *A*: heatmap: top 15 most overexpressed transcripts in each subgroup in normoxia. Rows = transcript expression in the corresponding subgroups (columns). *B*: dot plot: average transcript expression in each subgroup. Perforated blue lines = subgroups expressing genes like Schyns’ clusters 1 (C_1) and 2 (C_2) (25). Perforated green lines = subgroups expressing Cd206 and Lyve1. *C*: grouped UMAP plot with groups in normoxia (SL). *D*: G5 pathway analyses heatmaps. *Di*: functions *Dii*: canonical pathways. *Diii*: upstream regulators. Gray = N/A. *E*: bar graphs depicting the relative expression of Nlrp3, Osm, and S100a4. The error bars indicate the mean and standard error of the mean (SEM). *Ei*: single-cell RNA sequence expression data from normoxia [sea level (SL) ∼21% $\text{F}{\text{I}}_{{\text{O}}_{2}}$] (*n* = 1 mouse; *n* = 374 IMs), *day 1* (*D1*, *n* = 2 mice; *n* = 2,526 IMs), and *day 7* (*D7*, *n* = 2 mice; *n* = 1,211 IMs) post hypoxia exposure [5,486 m (hypoxia = ∼10% $\text{F}{\text{I}}_{{\text{O}}_{2}}$)]. *Eii*: RT-PCR (in biological triplicates) gene validation from SL, *n* = 3 (1 male and 2 females); *day 1*, *n* = 3 (1 female and 2 males); and *day 7*, *n* = 2 (1 male and 1 female). N/A, not applicable; SL, sea level; UMAP, uniform manifold approximation and projection.

We used heatmaps (Fig. 2*A*) to determine that subgroups in normoxia were distinct. We used IM markers previously described by Schyns et al. (25; Fig. 2*B*) to determine if our findings were consistent with previous reports. Our analyses revealed that subgroups 0 (SL_0) and 1 (SL_1) were both *H2-Eb1*(histocompatibility 2, class II antigen E beta)^hi^(CD74 antigen (invariant polypeptide of major histocompatibility complex, class II antigen-associated)^hi^/(mannose receptor, C type 1)^lo^Lyve1 (lymphatic vessel endothelial hyaluronan receptor 1)^−^ (Fig. 2*B*), similar to cluster 1 reported by Schyns et al. (25) and the IM3 population described by Gibbings (26). Subgroup 2 (SL_2) was H2-Eb1^lo^Cd74^lo^/Cd206^hi^Lyve1^hi^/Folr2 (folate receptor 2 (fetal))^hi^Cd163 (CD163 antigen)^hi^ (Fig. 2*B*), similar to cluster 2 reported by Schyns et al. (25) and the IM1/IM2 populations described by Gibbings (26). Subgroup 3 (SL_3) was H2-Eb1^lo^Cd74^lo^/Cd206^lo^Lyve1^−^. It was an intermediate population like subgroups SL_0 and SL_1 and subgroup SL_2 (Fig. 1*B*) but an offspring of a different node (Fig. 1*Cii*).

### The Largest Group in Normoxia Is Associated with Pattern-Recognition Receptors and the NLRP3-Inflammasome Complex

Binary hierarchical clustering of normoxia IMs revealed that SL_0 and SL_1 were similar and were grouped into G5, SL_2 into G3 and SL_3 into G1 (Fig. 1, *B* and *C*). G1 was small and invariant across hypoxia exposure time points (Fig. 1*Ciii* and Fig. 2*C*). G3 and G5 were the predominant NRCTs that coexist in the mouse lung, consistent with previous reports (Fig. 1*Ciii* and Fig. 2*C*; 25, 26).

We further investigated G5 because it was the most abundant (Fig. 1*Ciii* and Fig. 2*C*). Subgroups in G5 included SL_0 and SL_1 at normoxia, subgroup 1 at *D1* (D1_1), and subgroup 0 (D7_0) and 1 at *D7* (D7_1), which were 23% similar (Fig. 1*Ciii*). Examination of the functions across different hypoxia exposure time points varied, revealing subgroup phenotype heterogeneity (Fig. 2*D*). For example, at *D1*, the D1_1 subgroup was enriched with organismal death but not at normoxia (Fig. 2*Di*). Although G5 was over-represented with the role of pattern-recognition receptors in bacteria and viruses pathway (RPRRBV) at all time points, it was activated in D7_0 and inhibited in D7_1 (Fig. 2*Dii*). The RPRRBV pathway uses pattern-recognition receptors (PRRs) to recognize pathogen-associated molecular patterns (27). In this pathway, Nlrp3 [NACHT (NAIP (neuronal apoptosis inhibitor protein), C2TA (class 2 transcription activator, of the MHC), HET-E (heterokaryon incompatibility), and TP1 (telomerase-associated protein 1); LRR (leucine-rich repeat); and PYD (PYRIN domain)) domains containing protein 3], associated with the NLRP3-inflammasome complex, a sensor of cell injury patterns (27), was overexpressed in normoxia (log_2_ foldchange = 0.292). At *D1*, Nlrp3 was reduced (Fig. 2*Diii*), oncostatin M (Osm), an inflammatory cytokine in the RPRRBV pathway, was upregulated (log_2_ foldchange = 0.342), and apoptosis signaling appeared in hypoxia but not in normoxia. At *D7* in D7_0, Osm was downregulated (log_2_ foldchange = −0.493), but D7_1 was not enriched with cytokines (Fig. 2*Dii*). Because Nlrp3 and Osm play a functional role in the RPRRBV pathway, we validated their expression in individual scRNAseq data sets (Fig. 2*Ei*) using RT-PCR. Results revealed that the expression of Nlrp3 appeared high in normoxic cells. It decreased after *D1* and went back up at *D7* (Fig. 2*Eii*). On the other hand, the expression of Osm seemed to increase with the length of hypoxia exposure (Fig. 2*Eii*). We also validated one of G5’s marker genes, the S100A4 (S100 Calcium Binding Protein A4), which was highly expressed in most subgroups (Fig. 1*D*) and found that its expression appeared higher in hypoxic compared with normoxic conditions (Fig. 2*Eii*).

Among regulators related to the RPRRBV pathway, apolipoprotein E (Apoe), an endogenous pulmonary danger cue that primes and turns on the NLPR3 inflammasome in macrophages to produce IL-1β (28), was only activated in hypoxic conditions (Fig. 2*Diii*). Myeloid differentiation factor 88 (Myd88), an innate immune signal transduction adaptor, and interleukin 1 beta (IL-1β ), a potent proinflammatory cytokine, were active in normoxia but inhibited in hypoxia (Fig. 2*Diii*). Our previous study observed that at *D14*, Myd88 continued to be inhibited in hypoxia (7). These results confirm the changes in gene expression between normoxia and hypoxia exposure time points. Intracellular Toll-like receptor (TLR)-7 (TLR7) and TLR3 and cell surface TLR4 were inhibited at *D1* (Fig. 2*Diii*). Myd88 is an adaptor for inflammatory signaling pathways downstream of the interleukin-1 (IL-1) receptor family and Toll-like receptors (TLRs; Ingenuity Systems, http://www.ingenuity.com).

### On *Day 1*, Posthypoxia, the Most Abundant Group, G4, Was Associated with IL-1β and Osm Regulation

**Figure 3.**
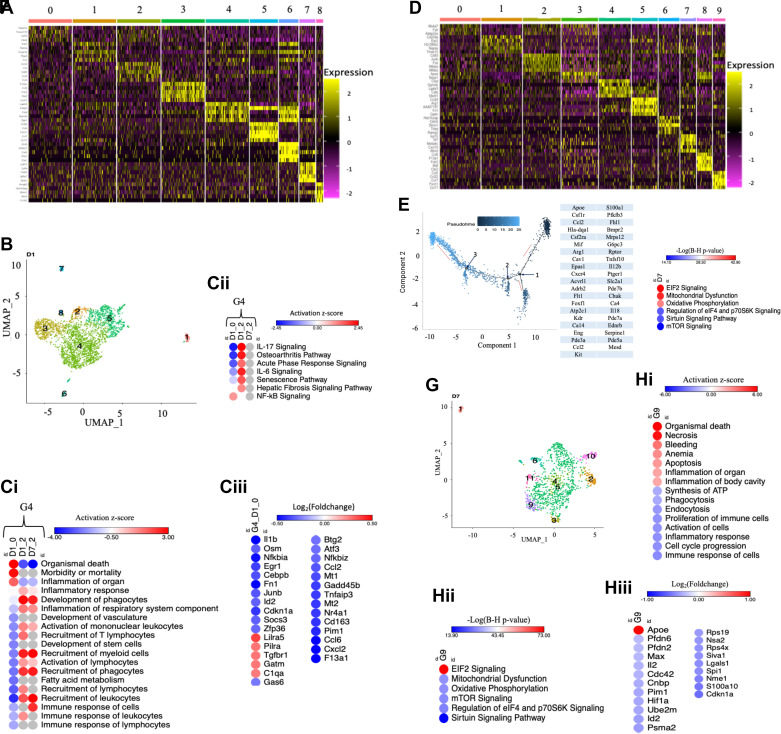
Five hypoxia-associated cell types appeared in response to hypoxia at *D1* and three at *D7*. *A*: heatmap: top five most overexpressed transcripts in each subgroup at *D1*. *B*: grouped UMAP plot depicting groups at *D1*. *C*: G4 pathways analyses heatmaps. *Ci*: functions. *Cii*: canonical pathways. *Ciii*: upstream regulators. *D*: top five most overexpressed transcripts in each subgroup at *D7*. *E*: cell trajectories and transcripts that vary according to the sequence of cellular states implicated in PH development. Dark blue = the beginning of the trajectory. Light blue = end of the trajectory. Red arrows = direction of the trajectory. *F*: pathways associated with transcripts that vary according to the sequence of cellular states. *G*: grouped UMAP plot depicting groups at D7. *H*: G9 pathway analyses heatmaps. Key for heatmaps: *Ci* and *Hi*: functions. *Cii* and *Hii*: canonical pathways. *Ciii* and *Hiii*: upstream regulators. Gray dots = not applicable. PH, pulmonary hypertension; UMAP, uniform manifold approximation and projection.

Eight groups appear in response to hypoxia at *D1* (Fig. 1*Ciii*). Assessing subgroup heterogeneity within HACTs at *D1* showed that each was unique (Fig. 3*A*). Binary hierarchical clustering revealed five new groups (Fig. 2*C* and Fig. 3*B*). Examination of the functions across different hypoxia exposure time points varied revealing subgroup phenotype heterogeneity within G4 [subgroup 0 (D1_0) and 2 (D1_2) at *D1* and subgroup 2 at *D7* (D7_2); Fig. 1*D* and Fig. 3*C*, *i* and *ii*]. For example, D1_0 was enriched with organismal death but resolved at *D7* (Fig. 3*Ci*). Interleukin-17 (IL-17 ) and IL-6 signaling associated with pro-inflammatory cytokines were overrepresented at *D1* but not *D7* (Fig. 3*Cii*). Detailed investigations of G4_D1_0 revealed that it was controlled in part by IL-1β and Osm (Fig. 3*Ciii*).

### A Rare Group Enriched with Transcripts Implicated in PH Development and Perturbed Pathways Associated with the Production of Impaired Repair Responses and Proinflammatory Cytokines Emerges on *Day 7* of Hypoxia

Defining subgroup heterogeneity at *D7* revealed that each was unique (Fig. 3*D*). Reasoning that *D7* represents a transition between acute and sustained hypoxia-induced responses, we ordered cells at *D7* into trajectories to determine transcripts that vary according to the sequence of cellular states and remove masking present in aggregating cells into clusters (Fig. 3*E*). The analyses identified 41 candidate transcripts implicated in PH development (Fig. 3*E*). In evaluating the pathways enriched with these transcripts, at the top was EIF2 signaling (Fig. 3*F*). It is involved in regulating the expression of proinflammatory cytokines (29). Mitochondria dysfunction was also overrepresented (Fig. 3*F*). It is associated with the production of proinflammatory cytokines and impaired repair responses (30). In our previous study, these pathways were perturbed at *D4* and continued at *D14* (7).

Three rare groups (G9, G10, and G11) unique to *D7* emerged (Fig. 3, *B* and *G*). G9 was the most abundant among the three (Fig. 3*G*). It was enriched in pathways associated with activating organismal death, inflammation, apoptosis, necrosis, and immune and inflammatory response inhibition (Fig. 3*Hi*). Pathway analysis revealed that EIF2 signaling, mitochondria dysfunction, and oxidative phosphorylation were also among the top pathways overrepresented in G9, similar to pathways enriched with transcripts that vary according to the cellular state (Fig. 3, *E**–Hii*), suggesting an association between G9 with transcripts involved in PH development. Regarding upstream regulators, most were downregulated except Apoe (Fig. 3*Hiii*).

### Cell Dynamics: There Is an Interplay between the Loss of Cells from Normoxia-Related Groups and the Emergence of Hypoxia-Associated Groups

**Figure 4.**
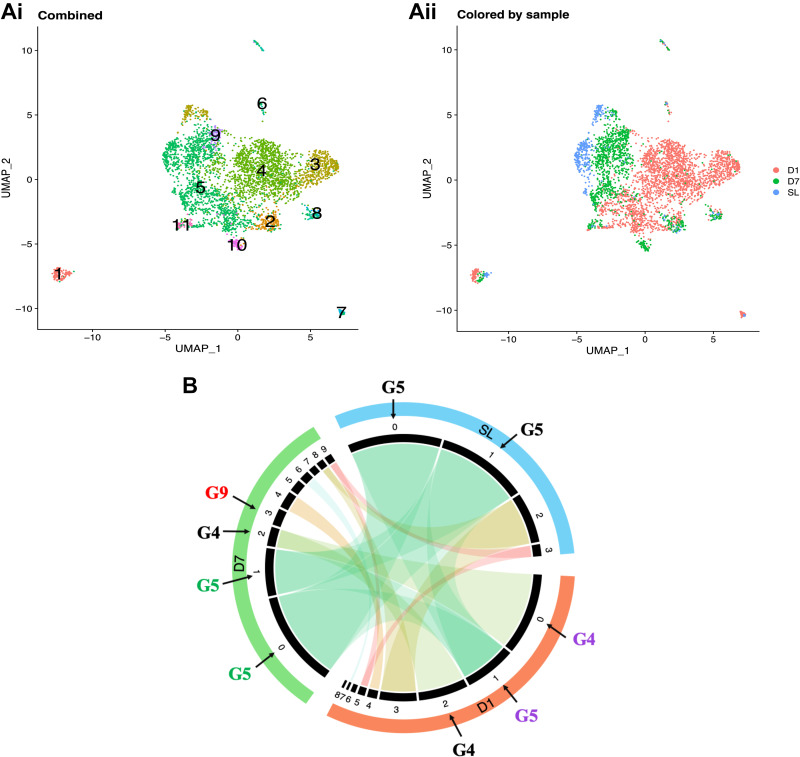
Proposed schematic of the macrophage disappearance reaction and niche model in IMs. *Ai*: group combined UMAP plots. Groups are colored the same as in Fig. 2*C* and Fig. 3, *B* and *G*. *Aii*: UMAP plots are colored by sample. *B*: circos plot represents percentages and similar subgroups across experimental time points. Black sectors = subgroups, sector width = subgroup cells proportion. Cords link matched subgroups. Cord transparency = similarity of the matched subgroups, the darker the clarity, the more similar. Purple = groups with unhealthy cells. Black arrows = cell percentages that add up to 75% at each experimental time point. Red = groups with unhealthy cells associated with transcripts implicated in PH development. PH, pulmonary hypertension; UMAP, uniform manifold approximation and projection.

Finally, we investigated cell dynamics by comparing group distributions across time points, focusing on the most abundant groups that emerged at each time point. To confirm matching results, groups with the same color from all time points clustered together in the combined sample (Fig. 2*C*, Fig. 3, *B* and *G*, and Fig. 4*Ai*). Overall, at *D1*, 62% of cells differed from those present in normoxia (Fig. 1*Ciii* and Fig. 4*Aii*). They included cells from subgroups 0, 2, 4, 6, 7, and 8 (Fig. 4*B*). However, after *D7*, only 34% were different (Fig. 1*Ciii* and Fig. 4*Aii*). They included cells from subgroups 2, 3, 4, 5, 6, and 7 (Fig. 4*B*).

Phenotype heterogeneity in G5 was also evident in the cell population dynamics. G5 represented 75% of the normoxic IMs and consisted of two subgroups (Fig. 4*B*). However, at *D1*, it converged (D1_1), and its population with an apoptosis-related insult dropped to 21%, representing a 54% decrease (Fig. 1*Ciii*, Fig. 2*Dii*, and Fig. 4*B*). In parallel, G4 emerged, with 54% IMs (Fig. 1*Ciii* and Fig. 4*B*). It was enriched with inflammation and immune response-related insults (Fig. 3*Ci*). Together G4 and G5 made up 75% of the population at *D1* (Fig. 1*Ciii* and Fig. 4*B*).

At *D7*, G5 diverged (D7_0 and D7_1), its population increased to 60% (Fig. 1*Ciii* and Fig. 4*B*), and organismal death was no longer activated (Fig. 3*Ci*). Conversely, G4 converged (D7_2), and its population dropped to 8% (Fig. 1*Ciii* and Fig. 4*B*). Organismal death resolved (Fig. 3*Ci*), becoming more like G5 (Fig. 3*G*). G9, containing 7% of the IMs, emerged (Fig. 1*Ciii* and Fig. 4*B*). It was controlled in part by Apoe (Fig. 3*Hiii*), the most significant gene implicated in PH development (Fig. 3*E*). G4, G5, and G9 populations added up to 75% (Fig. 1*Ciii* and Fig. 4*B*), and cells from G5 became more like those in normoxia (Fig. 4, *Aii* and *B*).

These results suggest an interplay between the loss of cells from G5 and the emergence of HACTs, which resemble resident-trained innate immunity macrophages (31, 32).

## DISCUSSION

We began by determining IM heterogeneity and its relationship with hypoxia-induced PH, postulating that scRNAseq and binary hierarchical clustering (BHC) could resolve IM diversity and its potential link to hypoxic PH. We demonstrated the existence of three predominant normoxia-related IM populations, confirming past findings (25, 26) and their continued presence following exposure to hypoxia. Aided by data generated from BHC, we report, for the first time, the existence of a putative resident-trained innate immunity IM population. It demonstrated a drive in the local environment to maintain a significant portion of the IMs (@75%) in a phenotype similar to that in normoxic homeostatic conditions, a process termed by others as resident-trained innate immunity (31, 32).

### Putative Resident-Trained Innate Immunity Macrophages Are Partly Depleted in the Initial Response to Hypoxia

When pattern recognition receptors (PRRs) expressed on the surface of macrophages were identified, the dogma of nonspecific innate immunity changed (31). It was found that they promote adaptive traits to safeguard against reinfection by pathogens, independent of the classical adaptive immune memory (31, 32).

Our study identified a population herein described as G5, associated with the RPRRBV pathway. This pathway is related to the innate immune system that uses PRRs to recognize pathogen-associated molecular patterns. PRRs are transmembrane receptors related to the NLRP3-inflammasome complex, a sensor of cell injury, and an interleukin (IL-1β)-processing platform that plays a role in the maturation and secretion of IL-1β from cells (27). IL-1β generates inflammatory cytokines during an immune response (33).

PRRs can be activated by endogenous molecules that do not have a microbial origin, resulting in sterile inflammation (34). When inhibited, PRR-mediated innate immune responses increase the risk of disease (35). The right amount of inflammasome activation is necessary for the host to cope with tissue damage, but abnormal activation might induce disease (36). These observations align with our findings: NLRP3 was present in normoxia, but it was neither activated nor inhibited.

At D1, G5, in response to hypoxia stress, decreased from 75% to 21%, suggesting a macrophage disappearance reaction (MDR) in which macrophages obliterate the insult and alert the immune system (37). Osm, an inflammatory cytokine in the RPRRBV pathway, was upregulated. However, MYD88, TLR3, TLR4, TLR9, and NLRP3 were inhibited, suggesting an inflammatory stimulus with an inhibited NLRP3 inflammasome. Concomitantly, G4 emerged with hypoxia. It made up 54% of the population, correlating with the loss of cells from G5. It was enriched with inflammation. However, IL-1β and Osm were downregulated. Recruitment of immune cells was inhibited, suggesting perturbations to the NLRP3 inflammasome. By *D7*, 46% of G4 disappeared, leaving 8% more related to G5, suggesting that G4 was transient, mainly in acute hypoxic conditions. G4 and G5 made up 75% of the IMs.

### Putative Resident-Trained Innate IMs Regenerate, and Macrophages with Transcripts Implicated in PH Development Emerge at *D7*

When resident macrophages (RMs) are depleted during an inflammatory stimulus, macrophages from circulating monocytes or self-proliferating RMs repopulate the space, an event termed the “niche model” (9). How much of the niche is replaced depends on the degree of inflammation (9). All RMs are depleted and replaced by bone marrow-derived monocytes during severe inflammation. Mild inflammation retains some cells and triggers lower recruitment of monocytes, which differentiate into highly proliferating immature cells (10).

In our study, G5 regenerates from 21% at *D1* to 60% at *D7*, aligning with the self-proliferation phenomenon (10, 38). Moreover, G9 representing 7% of the IMs, emerged associated with transcripts involved in PH development. Apoe is an endogenous pulmonary danger cue that primes and turns on the “NLPR3” inflammasome in macrophages to produce IL-1β (28), and the most significant gene implicated in PH development regulates it. Combining G9 with the 8% from G4 and 60% from G5, the populations added up to 75%. This observation parallels the idea that mild inflammation triggers decreased recruitment of monocytes (10) enriched with inflammatory monocytes, which differentiate into pathogenic macrophages (9). Of note, findings from the pathway analyses need further validation in future studies.

Thus, in normoxia, G5 is homogenous. But, at *D1*, G4 occupies 54% of its niche, and at *D7*, G4 (8%) and G9 (7%) occupy 15%. Although G5 is smaller (60%) at *D7* than in normoxia (75%), its niche is more diverse because G4 and G9 occupy 15%.

### Strengths and Limitations

We integrated scRNAseq and highlighted the transcriptome dynamics and BHC that quantified comparisons between the stimulus and control (12, 33, 39) to determine IM diversity and its association with PH (12, 33, 39). However, the small sample size across time points might have affected the effect size, limiting the detection of small differences between samples, a limitation common to many single-cell RNAseq studies (40). To address this limitation, we validated genes using RT-qPCR across time points to confirm their presence. We also compared groups in normoxic conditions to those already published to establish reproducibility and consistency (25, 26). In scRNAseq experiments, the unit of measurement is the cell and not the mouse, which increases the data points at each time point (16, 40).

### Conclusions

We found both normoxia-related and hypoxia-associated cell types in mouse lung IMs after exposure to hypoxia in vivo. We describe the existence of a putative resident trained innate immunity IM present in normoxia, transiently depleted, which recovers during sustained hypoxia. This cell type might act in concert with the macrophage disappearance reaction and niche model mechanisms to maintain cell phenotypes similar to those in normoxic homeostatic conditions (9). We also describe a rare putative pathogenic population associated with transcripts implicated in PH development that emerges in prolonged (7 days) hypoxia. These results shed light on cellular determinants of remodeling in the early stages of hypoxia-induced PH.

## ETHICAL APPROVAL

We used guidelines for animal experimentation established and approved by the Institutional Animal Care and Use Committees of the University of Colorado Anschutz Medical Campus to perform all mice procedures.

## DATA AVAILABILITY

Code for the pipeline used in the Seurat and ClusterMap analyses is available on GitHub at https://github.com/Iancam. CellRanger 2.1.1 was run with default settings, and the mm10 reference provided by 10X Genomics. The data supporting this study’s findings are openly available in the National Center for Biotechnology Information Sequence Read Archive (SRA) at https://www.ncbi.nlm.nih.gov/sra/PRJNA843087, accession number PRJNA843087.

## GRANTS

This work was supported by American Heart Association (AHA)-19CDA34730030, American Thoracic Society (ATS) /Pulmonary Hypertension Association (PHA) and Cardiovascular Medical Research and Education Fund (CMREF) Research Fellowship (to R.K.), National Institutes of Health (NIH) R25HL146166 (to C.M.), NIH NHLBI K25HL133481 (to V.O.K.), R01HL135872 (to B.G.), NIH T32HL007171 (to N.V.C), Department of Defense PR191774 (to K.R.S.), Department of Defense PR181125 (to K.R.S.), NIH P01HL014985 (to K.R.S.), and NIH P01HL152961 (to K.R.S.).

## DISCLOSURES

No conflicts of interest, financial or otherwise, are declared by the authors.

## AUTHOR CONTRIBUTIONS

N.V.C., C.M., S.K., H.Z., I.L.C., A.E.G., C.O.T., K.D., B.G., V.O.K., S.G., R.K., T.P., R.D.B., B.B.G., and K.R.S. conceived and designed research; C.M., H.Z., C.O.T., K.D., and B.G. performed experiments; N.V.C., I.L.C., and A.E.G. analyzed data; N.V.C. and K.R.S. interpreted results of experiments; N.V.C. prepared figures; N.V.C., and K.R.S. drafted manuscript; N.V.C., C.M., S.K., H.Z., I.L.C., A.E.G., C.O.T., K.D., B.G., V.O.K., S.G., R.K., T.P., R.D.B., B.B.G., and K.R.S. edited and revised manuscript; N.V.C., C.M., S.K., H.Z., I.L.C., A.E.G., C.O.T., K.D., B.G., V.O.K., S.G., R.K., T.P., R.D.B., B.B.G., and K.R.S. approved final version of manuscript.
